# Urinary tract infections due to *Trichosporon spp*. in severely ill patients in an intensive care unit

**DOI:** 10.5935/0103-507X.20150045

**Published:** 2015

**Authors:** Maria das Graças Silva Mattede, Cláudio Piras, Kelly Dematte Silva Mattede, Aline Trugilho Ferrari, Lorena Simões Baldotto, Michel Silvestre Zouain Assbu

**Affiliations:** 1 Department of Surgery, Escola Superior de Ciências, Santa Casa de Misericórdia de Vitória - Vitória (ES), Brazil.; 2 Department of Surgery, Universidade Federal do Espírito Santo - Vitória (ES), Brazil.

**Keywords:** Urinary tract infections/ epidemiology, Trichosporon, Critical illness, Intensive care units

## Abstract

**Objective:**

To evaluate the incidence of urinary tract infections due to *Trichosporon spp*. in an intensive care unit.

**Methods:**

This descriptive observational study was conducted in an intensive care unit between 2007 and 2009. All consecutive patients admitted to the intensive care unit with a confirmed diagnosis were evaluated.

**Results:**

Twenty patients presented with urinary tract infections due to *Trichosporon spp*. The prevalence was higher among men (65%) and among individuals > 70 years of age (55%). The mortality rate was 20%. The average intensive care unit stay was 19.8 days. The onset of infection was associated with prior use of antibiotics and was more frequent in the fall and winter.

**Conclusion:**

Infection due to *Trichosporon spp*. was more common in men and among those > 70 years of age and was associated with the use of an indwelling urinary catheter for more than 20 days and with the use of broadspectrum antibiotics for more than 14 days. In addition, patients with urinary infection due to *Trichosporon spp*. were most often hospitalized in intensive care units in the fall and winter periods.

## Abstract (pt)

**Objetivo:**

Avaliar a incidência de infecções do trato urinário por *Trichosporon spp*. em uma unidade de terapia intensiva.

**Métodos:**

Estudo descritivo observacional realizado em uma unidade de terapia intensiva no período de 2007 a 2009. Foram analisados todos os pacientes consecutivos que internaram na unidade de terapia intensiva e tiveram o diagnóstico confirmado.

**Resultados:**

Vinte pacientes apresentaram infecções do trato urinário por *Trichosporon spp*. A prevalência foi maior no sexo masculino (65%) e na faixa etária superior a 70 anos (55%). A mortalidade foi de 20%. A média de permanência na unidade de terapia intensiva foi de 19,8 dias. Seu aparecimento esteve relacionado ao uso pregresso de antibióticos e foi mais frequente no período que compreendeu o outono e o inverno.

**Conclusão:**

A infecção por *Trichosporon spp*. predominou no sexo masculino, de idade acima de 70 anos, com uso de sonda vesical de demora por mais de 20 dias e com uso de antibióticos de amplo espectro acima de 14 dias. Os pacientes que apresentaram a infecção urinária por *Trichosporon spp.* ficaram internados nos setores de terapia intensiva, com maior frequência, no período de outono e inverno.

## INTRODUCTION

Urinary tract infections and their complications are frequently found in medical practice, particularly among severely ill patients. With regard to the etiological agents of urinary tract infections, bacterial species are prevalent, particularly among hospitalized patients who use urinary catheters.^(1)^ Urinary tract infection affects patients of all ages, ethnicities, and sexes.^(2)^ Previous studies have shown that approximately 50% of women may experience at least one episode of either community- or hospital-acquired urinary tract infection in their lifetime.^(3)^

At present, with the routine use of cultures among patients in intensive care units (ICU), less common pathogenic organisms, such as yeast fungi, have emerged. These organisms include *Candida* and *Trichosporon*, which may also be involved in the pathogenesis of urinary tract infection in severely ill patients.^(4-6)^ Yeasts from the genus *Trichosporon* are considered emerging pathogens in hospitalized patients.^(7)^

An epidemiological study on the frequency of urinary tract infections in ICU patients has identified *Candida* *sp* as the most frequent pathogen, representing 28% of cases, followed by gram-negative bacilli, which include *Klebsiella pneumoniae*, *Escherichia coli*, and *Pseudomonas aeruginosa*.^(8)^

Fungal pathogens are responsible for severe infections and include yeasts of the genus *Trichosporon*.^(9,10)^

Fungal infections due to *Trichosporon* are frequently classified as superficial mycoses, are considered benign, and preferentially affect the scalp, armpits, and pubic region. In most cases, health professionals (physicians and nurses) involved in patient care may not be aware of this type of disease because the pathogen remains in intimate regions of the body.^(11)^

However, it has been observed that *Trichosporon* may cause systemic infections in humans, including urinary tract infections. In recent years, the incidence of hospital-acquired infection due to this fungus has increased, particularly among patients who are severely ill, who are immunosuppressed, who have prolonged hospital stays, and who undergo invasive procedures.^(12)^

The analysis of the predisposing factors for urinary tract infections among men indicates that longer urethral length, higher urinary flow, and the prostatic antibacterial factor are protective factors against ascending urinary tract infections compared with those that occur in women. However, in situations involving hospitalization associated with the use of urinary catheter, there may be a greater susceptibility to urinary tract infections caused by opportunistic microorganisms, primarily those that are resistant to antibiotics.^(13)^

Because of the increased presence of *Trichosporon* as an emerging pathogen among fragile and potentially immunosuppressed individuals with various diseases, together with the potential of these microorganisms to trigger severe and potentially lethal infections, the present study aimed to retrospectively evaluate urinary tract infections due to *Trichosporon spp*. among severely ill patients in ICU.

## METHODS

This observational descriptive cohort study was based on a retrospective analysis of medical records and was conducted between 2007 and 2009. The study population consisted of patients with urinary tract infection due to *Trichosporon spp*. who were hospitalized in the ICU and Recovery Center of* Santa Casa de Misericórdia de Vitória*, state of Espírito Santo, Brazil and who used antibiotic therapy and an indwelling urinary catheter. Patients without adequate data in their medical records were excluded. This study was approved by the Research Ethics Committee of the School of Medicine of the *Santa Casa de Misericórdia de Vitória *under protocol number 184/2009, and the requirement of informed consent was waived.

The parameters evaluated included urine cultures with *Trichosporon spp*. identification, clinical results compatible with urinary tract infection, and patient progression. The data collected in the forms included age, sex, duration of use of urinary catheters, use of antibiotics, types of antibiotics used, and the presence of *Trichosporon spp.* The microbiological analysis of urine culture and urinalysis type 1 was confirmatory and supported the continuation of the study.

The urine collection was standardized according to the recommendation of the Nosocomial Infection Commission and followed the following standard procedure: close the clip in the proximal region of the catheter; wait 15 to 30 minutes; collect urine using a sterile syringe, needle, and bottle using aseptic techniques and standard antisepsis; and immediately send the collected material to the clinical analysis laboratory. The microbiological analysis involved pathogen identification and evaluation of the sensitivity profile. Urinalysis type 1 involved the analysis of general aspects, abnormal elements, and sediment. Urine culture was performed using the standard method of culture by dilution with a calibrated loop of 0.001mL or 1µL. Dilution followed the traditional diagnostic criteria, which defines a cell count above 100 CFU/mL of urine as the limit indicative of urinary infection, particularly in women. For this study, Sabouraud glucose agar was used for fungal identification, in addition to blood agar media, which are frequently used for the study of other microorganisms that may be present in urine and may cause urinary tract infections. All plates were incubated for 24 - 72 hours at 35 - 37ºC and were examined every 12 hours to monitor colony growth.

Infection due to *Trichosporon spp.* was evaluated by quantitative analysis using colony count and by qualitative analysis. Qualitative analysis initially followed the pure culture criterion, i.e., no contamination with other microorganisms. Subsequently, qualitative macroscopic studies were conducted by evaluating colony morphology, and the evaluation of fungal structure was conducted via microscopy at 40x magnification using a direct technique and gram staining. For culture identification, manual tests, as well as tests on an NC 32 panel using a MicroScan autoSCAN-4 system (Siemens Healthcare^©^, Frankfurt, Germany), were performed. After culture analysis, urine samples were immediately sent to the urinalysis laboratory for type 1 urinalysis. The test was performed according to the recommendations of the National Health Surveillance Agency (*Agência Nacional de Vigilância Sanitária* - ANVISA) following the norms of the Brazilian Association of Technical Standards (Associação Brasileira de Normas Técnicas - ABNT).^(14)^

Upon receipt of the test results, which was always fewer than 5 days after sample collection, treatment was initiated via intravenous administration of 200mg fluconazole daily for 7 to 14 days.

## RESULTS

Of the 333 urine cultures evaluated, 20 (6%) were positive for *Trichosporon spp.*, of which 13 (65%) were found in male patients. Among the 20 patients with urinary tract infection due to *Trichosporon spp.*, 12 (60%) died. Positive cultures were more common among individuals > 70 years (55%).

The period between admission to the ICU and diagnosis of urinary tract infection due to *Trichosporon spp.* varied between 8 and 72 days, with most cases ranging from 10 to 30 days (75%), and with an average of 19.8 days. The average duration of use of an indwelling urinary catheter was 23.6 days.

The 20 infected patients exhibited nodules suggestive of white piedra in the armpits and pubic region, and this finding was used as a criterion for screening for fungus in the urine and was defined as a sentinel sign of infection onset.

With regard to seasonality, more infections (8 cases, 40%) were observed during winter.

All patients received antibiotic therapy prior to the fungal infection. The most commonly used antibiotics were fourth-generation cephalosporins (40%), quinolones (40%), carbapenems (30%), third-generation cephalosporins (30%), macrolides (20%), and other cephalosporins (70%). The results are summarized in table 1.

**Tabela 1:** Características dos pacientes com infecção por* Trichosporon spp.*

**Variáveis**	**N (%)**
Idade (anos)	
< 30	1 (5)
40 - 50	1 (5)
50 - 60	4 (20)
60 - 70	3 (15)
> 70	11 (55)
Tempo entre internação e diagnóstico da infecção (dias)	
< 10	3 (15)
10 - 15	9 (45)
16 - 30	6 (30)
> 30	2 (10)
Tempo do uso de sonda vesical de demora (dias)	
< 10	4 (20)
10 - 15	6 (30)
16 - 30	5 (25)
31 - 45	2 (10)
> 45	3 (15)
Estação do ano	
Primavera	4 (20)
Verão	2 (10)
Outono	6 (30)
Inverno	8 (40)
Antibióticos utilizados	
Carbapenêmicos	6 (30)
Quinolonas	8 (40)
Cefalosporinas	14 (70)
Cefalosporinas de 3^a^ geração	6 (30)
Cefalosporinas de 4º geração	8 (40)
Macrolídeos	4 (20)

## DISCUSSION

Our results indicate that the prevalence of *Trichosporon spp.* in urine cultures from severely ill patients in ICU is approximately 6% and is more common among men aged > 70 years (55%). A relevant observation was that all patients used antibiotics, particularly cephalosporins and quinolones, prior to infection.

In general, urinary tract infection in adults is more common among women. This increased susceptibility is due to anatomical conditions, i.e., shorter urethra that is in close proximity to the vagina and anus.^(15)^ However, this finding was not corroborated by the present study, as 65% of the infections occurred in men. Accordingly, a study conducted at the *Instituto do Coração* of the *Hospital das Clínicas* of the *Faculdade de Medicina* of the *Universidade de São Paulo* reported 24 urinary tract infections due to *Trichosporon spp.*, of which 71% occurred in men.^(7)^

The mortality rate reported in the literature from infections due to *Trichosporon spp.* is high and reaches 83%.^(7,16)^ Our results also indicate a high mortality rate, which is a cause for concern because this microorganism has a purely aesthetic importance in the dermatological literature and shows a low level of pathogenicity in healthy patients. However, the case series studied here comprised a larger number of patients aged > 70 years, and these individuals could possibly have chronic conditions associated with immunosuppression and increased susceptibility to atypical urinary infections.^(7)^ However, the study design does not allow us to infer that the deaths were caused by this pathogen.

*Trichosporon* species have been described as opportunistic agents that cause systemic disease in immune-compromised patients. The isolation of this microorganism in urine samples has been rarely described in the literature and is more frequent in older people.

In cases of prolonged hospital stay, patients undergo several different treatments, including antibiotic therapy and invasive procedures. This compromises the natural barriers of the skin and mucosa, increasing the risk of opportunistic infections and complications, including urinary tract infections.^(17)^ Epidemiological data indicate that the most prevalent nosocomial infection is urinary tract infection.^(2)^ In addition, 80% of the cases of urinary tract infection in ICU patients are associated with the use of indwelling catheters.^(18,19)^

A study from the 1970s involving a group of 98 patients observed yeasts in urine samples that presented an average of 12 days after the implantation of urinary catheters.^(4)^ Fungal development among patients using indwelling catheters is facilitated by the formation of biofilm, which could explain the persistence of infection with *Trichosporon spp.*, despite its *in vitro* sensitivity to antifungal agents.

The severity of the clinical course increases because microorganisms that form biofilms are more protected from the host’s immune system, can communicate by quorum sensing, and become resistant to most conventional antimicrobial agents used in the treatment of infections. These factors promote the progression to systemic infections by favoring the perpetuation of infectious foci, which become difficult to control with antimicrobial agents.^(20-23)^ In this study, the nodules observed in the patients’ hair served as signs for the diagnosis of urinary tract infection due to *Trichosporon spp.* and were considered sentinel signs for possible onset of infection.

Previous studies have not reported any correlation between infection and seasonality. However, the most frequent cases of urinary tract infection due to *Trichosporon spp.* occurred during the colder seasons. It is known that this disease has a cosmopolitan geographic distribution with higher prevalence in tropical and temperate climates, including South America and Middle East, and it is more rare in North America and Europe. With regard to the *in vitro* morphology, most *Trichosporon* cultures maintained at room temperature (25ºC) exhibit a coarse texture (64.3%) and a dry and opaque appearance. However, at 37ºC, these fungi exhibit a predominantly smooth texture (71.4%), a moist and shiny appearance (53.6%), and a cream color.^(24)^

The use of antibiotics in severely ill patients favors fungal development, particularly by yeasts, and the onset of opportunistic infections.^(19)^ Narrow- and broad-spectrum antibiotics have broader mechanisms of action against bacteria when used in combination, and they selectively favor fungal growth in hospitalized patients.^(8)^

In the treatment of hospitalized patients with a laboratory diagnosis of urinary tract infection due to *Trichosporon spp.*, the following criteria and clinical procedures have been adopted: immediate removal of the urinary catheter, sufficient hydration of the patient to maintain adequate diuresis, intravenous administration of 200 mg of fluconazole daily for 7 to 14 days, performance of type 1 urinalysis every 72 hours, monitoring of the general condition of the patient, and performance of a control urine culture after treatment. With regard to antifungal activity, Araújo Ribeiro et al.^(25)^ found variable sensitivity rates of *Trichosporon spp.* to amphotericin B (76%), fluconazole (81%), and caspofungin, micafungin, and anidulafungin (100%).

The limitations of this study include the fact it was conducted in a single center, the small sample size, and the purely descriptive nature of the study, which prevents evaluation of the risk factors for this infection and the establishment of a correlation between infection due to *Trichosporon* and mortality.

Therefore, standardization of the approaches to be used by clinicians and intensivists for the early detection of this etiological agent is essential to ensure appropriate and effective treatment of severely ill patients. This standardization is required in cases of colonization or infection by yeasts, including *Trichosporon spp.*, particularly among patients with urinary catheters.^(1,7,18,19,26)^

## CONCLUSION

Infection due to *Trichosporon spp*. was more common in men age > 70 years and was associated with the use of indwelling urinary catheter for more than 20 days and with the use of broad-spectrum antibiotics for more than 14 days.

Patients with urinary infection due to *Trichosporon spp.* were more frequently hospitalized in intensive care units in the fall and winter periods.
